# Heterotrophic cultivation of *Auxenochlorella protothecoides* using forest biomass as a feedstock for sustainable biodiesel production

**DOI:** 10.1186/s13068-018-1173-1

**Published:** 2018-06-20

**Authors:** Alok Patel, Leonidas Matsakas, Ulrika Rova, Paul Christakopoulos

**Affiliations:** Biochemical Process Engineering, Division of Chemical Engineering, Department of Civil, Environmental, and Natural Resources Engineering, Luleå University of Technology, 971 87 Luleå, Sweden

**Keywords:** *Auxenochlorella protothecoides*, Wood biomass, Organosolv pretreatment, Heterotrophic growth, Lipid production, Biodiesel

## Abstract

**Background:**

The aim of this work was to establish a process for the heterotrophic growth of green microalgae using forest biomass hydrolysates. To provide a carbon source for the growth of the green microalgae, two forest biomasses (Norway spruce and silver birch) were pretreated with a hybrid organosolv-steam explosion method, resulting in inhibitor-free pretreated solids with a high cellulose content of 77.9% w/w (birch) and 72% w/w (spruce). Pretreated solids were hydrolyzed using commercial cellulolytic enzymes to produce hydrolysate for the culture of algae.

**Results:**

The heterotrophic growth of *A. protothecoides* was assessed using synthetic medium with glucose as carbon source, where the effect of sugar concentration and the carbon-to-nitrogen ratio were optimized, resulting in accumulation of lipids at 5.42 ± 0.32 g/L (64.52 ± 0.53% lipid content) after 5 days of culture on glucose at 20 g/L. The use of birch and spruce hydrolysates was favorable for the growth and lipid accumulation of the algae, resulting in lipid production of 5.65 ± 0.21 g/L (66 ± 0.33% lipid content) and 5.28 ± 0.17 g/L (63.08 ± 0.71% lipid content) when grown on birch and spruce, respectively, after only 120 h of cultivation.

**Conclusions:**

To the best of our knowledge, this is the first report of using organosolv pretreated wood biomass hydrolysates for the growth and lipid production of microalgae in the literature. The pretreatment process used in this study provided high saccharification of biomass without the presence of inhibitors. Moreover, the lipid profile of this microalga showed similar contents to vegetable oils which improve the biodiesel properties.

## Background

Progressive depletion of fossil fuels, global climate change issues, and growing demand for energy have led to a move toward an alternative, renewable, and sustainable source of energy [1]. Among the various alternative energy sources, biofuels are a more sustainable option for transportation fuels and for the growing energy demands of industry. The worldwide biofuel market is still in its infancy [2]. In Europe, the policies relating to climate change and renewable energy are decided by the European Union (EU). The EU has indicated that research on biofuels production using various sources of biomass could play a vital role in replacing fossil fuels until 2045 [3]. According to the Swedish renewable energy policy, the local transport sector (exclusive of domestic aviation) should reduce the green house gas (GHG) emissions by 70% until 2030, compared to the emissions in 2010 [4]. There has been rapid progress in global biofuel production over the last decade. However, the first-generation biofuels have raised significant concerns due to their sustainability, as their production is based on vegetable oil and food crop sources that directly compete with sources of food for humans. The use of human food stocks for biofuel production is no longer cost-effective or ethical in the present situation [5]. In contrast to the first-generation biofuels, non-edible lignocellulosic biomasses such as agricultural and forestry residues are used as feedstocks for the production of the second-generation biofuels [2]. This type of fuel reduces the direct competition between food and fuel. However, this strategy has again raised an important question regarding cost-effectiveness, due to the inefficient process of conversion of biomass and the low energy efficiency [6]. To replace the direct conversion of lignocellulosic biomass to low-value fuel for the generation of heat and electricity, conversion to high-added value-quality bio-products and energy carriers has been proposed [7], aimed at the establishment of a bio-refinery concept. To achieve this, it is very important to make use of all the major components of lignocellulosic biomass, namely cellulose, hemicellulose, and lignin. In this scheme, the use of hybrid organosolv-steam explosion method allows the efficient fractionation of biomass, resulting in three isolated fractions that can be used in various processes.

Microbial oil sources as feedstock for the third-generation biofuels have many advantages over their counterparts. In the last decade, microalgae have emerged as a promising source for the production of renewable biofuels, as they are efficient photosynthesizers, their culture require less growth area than terrestrial plants, and they are able to channel most of their energy into cell division, which enhance biomass productivity [8]. They can be grown on infertile land, polluted land, or arid land, so they do not compete with the use of land for food production. Most of the algal species are oleaginous and can accumulate very high quantities of intracellular lipids (> 60% w/w lipid content) under various conditions of stress such as nutrient limitation. Moreover, they have a great ability to make use of municipal wastewater and industrial effluents, which makes them potential candidates for large-scale production [9]. Thus, biodiesel derived from lipids stored in oleaginous microalgae can reduce environmental pollution and it is a promising substitute for the conventional diesel. In addition, the biodiesel obtained from microalgae is eco-friendly, is devoid of any harmful toxic substances, and contributes less greenhouse gas emissions than fossil diesel fuel [8].

The main restriction to using oleaginous microalgae as feedstock for biodiesel production is their culture techniques. There are four cultivation techniques appropriate for microalgae, which can be divided into autotrophic, heterotrophic, mixotrophic, and photoheterotrophic modes of culture [10]. Microalgae use inorganic carbon in the form of CO_2_ and energy from sunlight to generate organic matter when cultured using the autotrophic mode [10]. The microalgal oil can be produced by sequestering of atmospheric CO_2_, which is the major advantage of this mode [11]. Microalgae solely depend on the exogenous organic carbon sources provided for their growth in heterotrophic cultivation, while, in mixotrophic cultivation, they use both light and exogenous organic carbon as energy sources [12]. However, in the photoheterotrophic mode of culture light is required to metabolize the external carbon (as in mixotrophic culture), but the major difference between these two modes is that carbon dioxide cannot be absorbed and metabolized as it can be in mixotrophic mode [13]. Heterotrophic culture has many advantages over its counterparts, as it is cost-effective and comparatively easy to operate with quite low daily maintenance. Moreover, heterotrophic cultivation can be performed in any fermenter without illumination; hence, there is no requirement of photobioreactor as in case of autotrophic cultivation which finally reduces the overall production cost [14]. A high quantity of microalgal culture (e.g., 100,000 L) grown in heterotrophic mode can generate almost 500 tons of dry biomass of *Chlorella* species, which is equivalent to 50% of the total Japanese production of this algae [15].

Glucose is the most common substrate used for heterotrophic culture of microalgae, but it should be obtained from renewable sources [10]. To become more economically viable and continue to exist in the biofuel market, heterotrophic cultivation must use inexpensive raw materials such as inedible lignocellulosic feedstocks. Different types of biomass have been used for the heterotrophic culture of microalgae such as rice straw, corn powder, cassava, Jerusalem artichoke, sugarcane, sweet sorghum, waste molasses, soy whey, and glycerol as a by-product of the biodiesel industry, which give more cellular biomass and lipid production compared to the use of pure glucose [16]. Sweden has a well-developed forestry industry that contributes substantially to its economy. It has a huge forest area, which covers approximately 53.1% of the total land area, involving 3490 million m^3^sk of total standing volume of forest [17]. Norway spruce (*Picea abies*), Scots pine (*Pinus sylvestris*), and birch (*Betula pendula* and *B. pubescens*) are the foremost tree species representing 40.8, 39.2, and 12.4% of the total standing volume of the forest, respectively [17]. The use of forest residues for biofuel production has attracted interest due to generation of additional revenue and the reduction of greenhouse gas emissions. In the present study, two different types of forest biomass, softwood (spruce) and hardwood (birch), were used as raw materials for the culture of green microalgae. These two lignocellulosic biomasses contain different concentrations of cellulose, hemicellulose, and lignin, and the complex structures of these substrates make them difficult to hydrolyze. A pretreatment step is required before hydrolysis of recalcitrant materials, to change the size and structure of lignocellulosic biomass [18], thus resulting in easy enzymatic hydrolysis of the complex carbohydrates to monomeric sugars. After selection of suitable feedstocks for the heterotrophic culture of microalgae, the selection of a suitable candidate microalga is crucial to utilize these hydrolysates efficiently for high lipid productivity. *Auxenochlorella protothecoides* formerly known as *Chlorella protothecoides* was selected for the current study, due to its robust characteristics. It is well adapted for heterotrophic culture and, as reported in the literature, and it can accumulate up to 60% w/w of lipid content in its cellular compartments when grown on various lignocellulosic hydrolysates, as listed in Table 1 [19–28]. However, to the best of our knowledge, it has not been reported the cultivation of *A. protothecoides* on hydrolysates from wood biomass.

**Table 1:** Assessment of various microalgae grown on different lignocellulosic biomass

Oleaginous microalgae	Cultivation medium (pretreatment/hydrolysis)	Cell dry weight (g/L)	Lipid content (%, w/w)	References
*C. pyrenoidosa*	Glucose (10 g/L)	0.92	50.3	[19]
	Rice straw hydrolysate (acid pretreatment and enzymatic hydrolysis)	2.83	56.3	
*C. pyrenoidosa*	Glucose	2.34	21.5	[20]
	Artificial mix sugars	4.23	31	
	Sugarcane bagasse hydrolysate (enzymatic hydrolysis)	5.8	34	
*C. protothecoides*	Glucose (10 g/L)	3.74	54.7	[21]
	Corn powder hydrolysate (enzymatic hydrolysis)	3.92	55.3	
*C. protothecoides*	Glucose (40 g/L)	10.7	30.7	[22]
	Cassava starch hydrolysate (two-step enzymatic hydrolysis)	15.8	26.5	
*C. vulgaris*	Hydrolysate from *Cyperus esculentus* waste (two-step enzymatic hydrolysis)	4.43	34.44	[23]
	Glucose (30 g/L)	1.85	22.89	
*C. protothecoides*	Glucose	3.39	47.7	[24]
	Cassava starch hydrolysate (hot water treatment followed by enzymatic hydrolysis)	4.26	50.2	
	Corn powder hydrolysate (enzymatic hydrolysis)	4.67	39.9	
*C. protothecoides*	Glucose (10 g/L)	3.7	53.3	[25]
	Fructose (10 g/L)	3.9	52.7	
	Sucrose (10 g/L)	1.2	37.7	
	Sweet Sorghum hydrolysate (acid hydrolysis in autoclave followed by enzymatic hydrolysis)	5.10	52.5	
*C. protothecoides*	Waste molasses hydrolysate N limited medium (enzymatic hydrolysis)	70.9	57.6	[26]
	Waste molasses hydrolysate direct medium (enzymatic hydrolysis)	97.1	57.1	
*C. protothecoides*	Sugarcane juice hydrolysate (enzymatic hydrolysis)	1.23	43.1	[27]
	Glucose (20 g/L)	1.43	46.7	
*C. protothecoides*	Jerusalem artichoke (enzymatic hydrolysis)	NA	45.2	[28]
*A. protothecoides*	Glucose (20 g/L) C/N;60	8.40	64.52	This study
	OPBH (C/N; 60) (organosolv-steam explosion followed by enzymatic hydrolysis)	8.56	66.00	
	OPSH (C/N; 60) (organosolv-steam explosion followed by enzymatic hydrolysis)	8.37	63.08	

Comparative analysis of the oleaginous microalga *A. protothecoides* grown on glucose and also on hydrolysates of various lignocellulosic biomass sources under heterotrophic mode of cultivation

## Results and discussion

### Effect of initial glucose concentration on the growth and lipid accumulation of the oleaginous microalga *A. protothecoides*

Among the various substrates that can be provided, glucose is a preferred carbon source for maintenance of the heterotrophic growth of oleaginous microalgae, as it has higher energy content per mol (~ 2.8 kJ/mol) than other substrates such as acetate (~ 0.8 kJ/mol) [29]. To determine the effect of the initial concentration of glucose on the cell dry weight and lipid accumulation in the oleaginous microalga *A. protothecoides*, five different concentrations of glucose ranging from 20 to 100 g/L were included in the basal medium (BBM), which contained yeast extract as nitrogen source. The C/N (g/g) ratio was kept at 20 for each flask and the flasks were inoculated with a 10% volume of exponentially growing seed culture.

Table 2 shows the results for cell dry weight, biomass yield, total lipid concentration, lipid yield, and lipid content for *A. protothecoides* grown on various initial concentrations of glucose. The highest cell dry weight (10.92 ± 0.32 g/L) was obtained when the microalgae were grown at 100 g/L glucose, but this was not much higher than the 8.18 ± 0.34 g/L cell dry weight which was obtained at 20 g/L glucose. Moreover, the biomass formation yield was lower at 100 g/L (0.319 ± 0.011 g/g_substrate_) than at 20 g/L (0.456 ± 0.008 g/g_substrate_). As shown in Table 2, cell dry weight was slightly lower (7.30 ± 0.48 g/L) at 40 g/L glucose and increased correspondingly as the glucose concentration increased. Approximately 8.22 ± 0.21, 9.54 ± 0.19, and 10.92 ± 0.32 g/L cell dry weight was obtained when glucose was kept between 60 and 100 g/L. These data suggested that the increasing concentration of glucose did not contribute to the biomass productivity, as some of the glucose that was observed at the time of residual glucose concentration determination by HPLC was not consumed (Table 2). This alga cultured on 20 g/L glucose showed an exceptional value of lipid yield per unit of glucose consumed; 0.150 ± 0.004 g/g_substrate_, compared to culture with a higher amount of glucose (Table 2). In the case of oleaginous microorganisms, the maximum theoretical yield of lipid produced per unit of glucose consumed is 0.33 g/g when cultured with glucose under nitrogen-limiting conditions, but, in most cases, the conversion values do not exceed 0.20 g/g [30]. However, it was interesting to note that the cells showed accumulation of lipid in an irregular manner regardless of their biomass formation. Approximately 1.68 ± 0.19, 1.28 ± 0.09, 1.58 ± 0.17, and 2.04 ± 0.23 g/L total lipid was obtained when the cells were grown on glucose concentrations of 40–100 g/L, respectively. The highest total lipid yield (2.70 ± 0.12 g/L) was obtained when the glucose concentration was 20 g/L, which is equivalent to 33 ± 0.52% w/w of the lipid content on a cell dry weight basis (Table 2). This concentration of glucose (20 g/L) was, therefore, selected for further experiments.

**Table 2:** Growth of *A. protothecoides* on various initial glucose concentrations

Initial glucose concentration (g/L) in GSM	Cell dry weight (g/L)	Lipid concentration (g/L)	Lipid content (%, w/w)	Biomass yield (g/g_substrate_)	Lipid yield (g/g_substrate_)	Residual glucose concentration (g/L)
20	8.18 ± 0.34	2.70 ± 0.12	33.00 ± 0.52	0.456 ± 0.008	0.150 ± 0.004	2.05 ± 0.07
40	7.30 ± 0.48	1.68 ± 0.19	23.01 ± 0.84	0.267 ± 0.016	0.061 ± 0.009	12.66 ± 0.17
60	8.22 ± 0.21	1.28 ± 0.09	15.57 ± 0.59	0.296 ± 0.007	0.046 ± 0.002	32.26 ± 0.23
80	9.54 ± 0.19	1.58 ± 0.17	16.56 ± 0.43	0.314 ± 0.006	0.052 ± 0.001	49.65 ± 0.41
100	10.92 ± 0.32	2.04 ± 0.23	18.68 ± 0.76	0.319 ± 0.011	0.059 ± 0.001	65.78 ± 0.37

Effect of different initial concentrations of glucose on the cell dry weight (g/L), total lipid concentration (g/L), lipid content (% w/w), biomass yield (g/g_substrate_), lipid yield (g/g_substrate_), and residual glucose (g/L) in *A. protothecoides* after 120 h of culture on glucose-synthetic media (GSM)

With the same oleaginous microalga *C. protothecoides*, it has been found that 3.7 g/L cell dry weight and 53.3% w/w lipid content could be obtained after 120 h of culture when the initial glucose concentration was 10 g/L [25]. In comparison, *C. protothecoides* has been found to give 0.92 g/L cell dry weight with 50.3% w/w lipid content after 60 h when grown on 10 g/L glucose [19]. In another work, this microalga produced 10.7 g/L dry biomass with 30.7% w/w of lipid content after 240 h of cultivation, when grown on 40 g/L glucose [22]. According to the above observations, the dry biomass and lipid content of microalgal species are not only dependent on the initial concentration of sugar but also vary with the other medium components and the culture conditions provided. The selection of suitable candidate organisms and optimization of the medium for their growth and accumulation of lipid are important factors for biodiesel production from oleaginous microorganisms, as these are the very first steps to achieving this goal. Several microalgal species are well known for producing specific classes of fatty acids in their cellular compartments through simple adjustment of their culture medium [31]. These unusual and valuable lipids from microalgal species have been shown to be a significant contribution to various industrial applications [32]. The synthesis of different groups of fatty acids in any oleaginous microorganisms (including microalgae) depends on various factors such as culture temperature; mode of culture (autotrophic, mixotrophic, or heterotrophic); concentration, and ratio of the carbon, nitrogen, and phosphorus sources; pH, and mainly the strain of the microalga [33]. It has been reported that *C. protothecoides* can synthesize four times as much lipid (57.9%, w/w) when it is grown under heterotrophic conditions than under autotrophic conditions [21, 34].

### Effect of various C/N (g/g) ratios on the accumulation of lipid in *A*. *protothecoides*

**Fig. 1:**
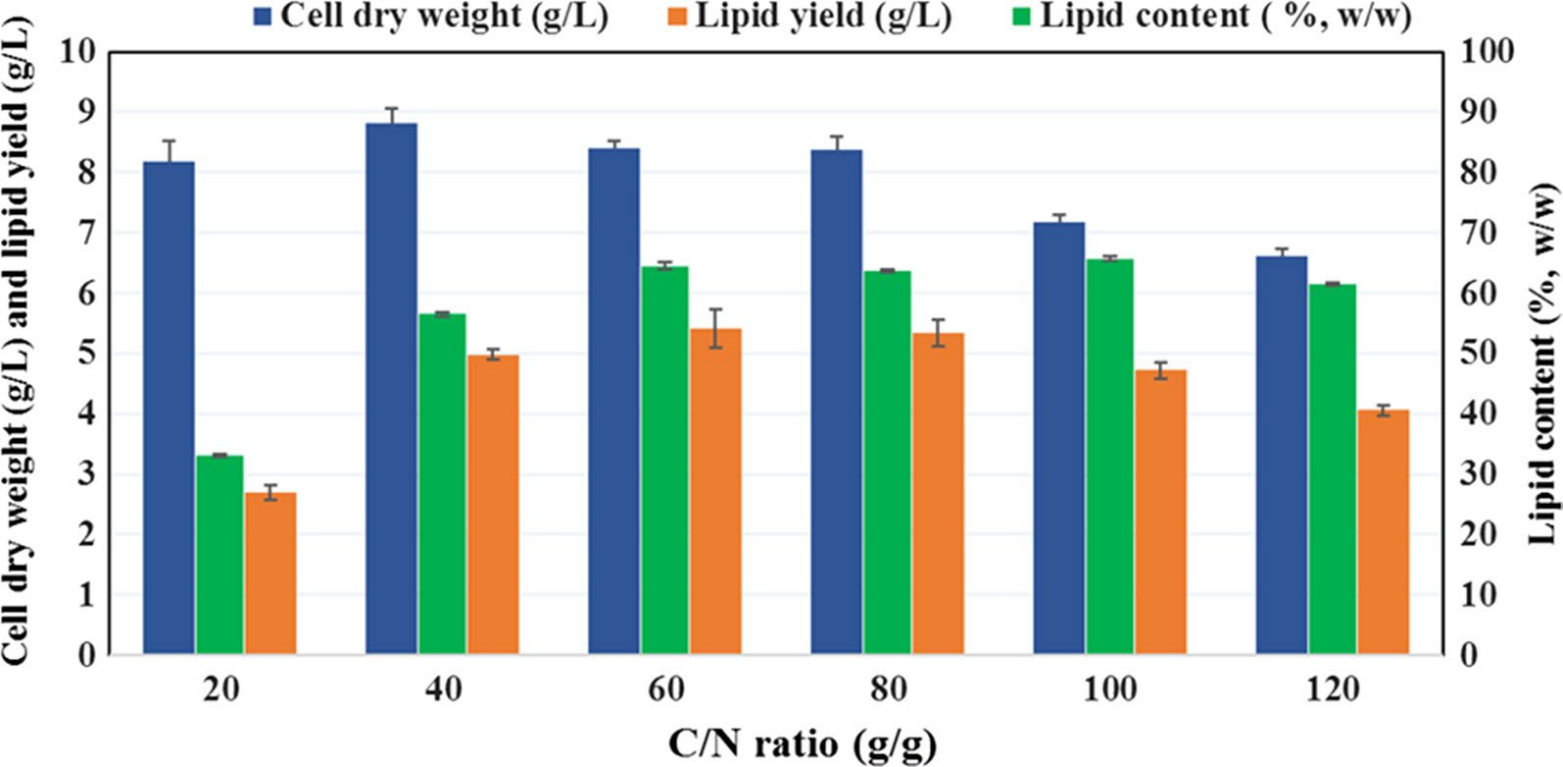
Assessment of growth and lipid accumulation of *A. protothecoides* in different C/N ratio. Effect of various C/N ratios on the cell dry weight (g/L), total lipid concentration (g/L), and lipid content (% w/w) in *A. protothecoides* after 144 h of culture

Lipid accumulation in the cellular compartment of oleaginous microorganisms is dependent on the metabolic accessibility of the nutrients provided. If an oleaginous microalga is grown under conditions of nutrient limitation with access to excess carbon, it shows greater lipid accumulation [35]. Thus, to study the lipid accumulation, the *A*. *protothecoides* cultures were supplemented with an excess of glucose by adjustment of the nitrogen concentration through alteration of the C/N (g/g) ratio, from 20 to 120. It has previously been reported that the components of medium such as carbon, nitrogen, phosphorus, and sulfur and their ratios (C/N, C/P, C/S) have a significant influence on growth and lipid accumulation in oleaginous microorganisms [36]. The cell dry weight (g/L), total lipid concentration (g/L), and lipid content (%, w/w) of *A*. *protothecoides* grown with different C/N ratios and with 20 g/L glucose are shown in Fig. 1. The cell dry weight initially increased with increasing C/N ratio up to a value of 40, whereas any further increase in C/N ratio resulted in a reduction in cell dry weight, which was more rapid for ratios above 100. The highest cell dry weight (8.82 ± 0.24 g/L) was obtained at a C/N ratio of 40. The lipid concentration increased significantly when the C/N ratio was increased from 20 to 60, but any further increase in this ratio resulted in a decline in lipid concentration. The highest total lipid concentration (5.42 ± 0.32 g/L) was observed at a C/N ratio of 60, with a corresponding lipid content of 64.52 ± 0.53% w/w. Based on the above, a C/N ratio of 60 was selected for the heterotrophic culture of *A*. *protothecoides* using lignocellulosic biomass hydrolysates as the carbon source. Microbial lipid content can be enhanced by changing the culture conditions, but of the various kinds of strategies, nitrogen starvation is the most prominent and convenient technique for enhancement of the lipid yield [37]. It has also been reported that a two-stage culture technique in which the microalgae are initially grown in a nitrogen-rich medium and then transferred to a nitrogen-limited medium could also offer an alternative to total nitrogen limitation. The first stage supports the high production of biomass; this is followed by stress caused by nitrogen limitation, which in turn induces the accumulation of lipid [38]. In general, any oleaginous microorganism channels excess carbon toward the accumulation of triacylglycerol under conditions in which a key nutrient (such as nitrogen) is exhausted. However, in a different study with some marine and freshwater species such as *Crypthecodinium cohnii* and *C. sorokiniana*, it was suggested that the lipid accumulation may not be due to exhaustion of a nutrient but also due to the excess carbon in the culture medium [39, 40]. The accumulation of lipid in oleaginous microorganisms is not only dependent on the lipid-synthesizing enzymes associated with nitrogen starvation but also on the cessation of other enzymes involved in cell growth and proliferation [41]. Because of this, under conditions of nutrient exhaustion, inhibition of cell division leads to a reduced growth rate, so the carbon is channelled toward the synthesis of high amounts of lipids in the intracellular compartments [33]. Under conditions of nitrogen limitation, a cascade of reactions is responsible for the formation of acetyl-CoA in oleaginous microorganisms [42]. Increased activity of AMP deaminase decreases the levels of the mitochondrial and cellular AMP, and blocks the function of isocitrate dehydrogenase for conversion of isocitrate to α-ketoglutarate. Isocitrate cannot be metabolized in the mitochondria, so it equilibrates with citrate via aconitase; citrate enters the cytosol from the mitochondria through an efficient citrate efflux system and is cleaved by ATP citrate ligase (ACL) to give acetyl-CoA and oxaloacetate. Acetyl-CoA enters the pathway for fatty acid synthesis. However, this is not the complete story for lipid synthesis in oleaginous microorganisms under nitrogen-limiting conditions [33]. After the carbon source, the quality and quantity of the nitrogen source is an important factor contributing to the high dry biomass and lipid yield of microalgal cells (except in diatoms, where silicon plays a more significant role than nitrogen) [43]. In general, a sufficient quantity of nitrogen has a positive effect on gaining high biomass but has a negative effect on lipid accumulation. Microalgae have the capacity to assimilate various nitrogen sources such as nitrate, ammonia, urea, yeast extract, and peptone with complex sources such as yeast extract having a potentially positive effect on growth and lipid accumulation, because they contain a mixture of amino acids, vitamins, and growth factors [44].

### Time course experiment with *A. protothecoides* grown on hydrolysates from organosolv-steam explosion pretreated birch and spruce biomass

Different factors, such as the crystallinity and the degree of polymerization of cellulose, the surface area accessible, and the quantity and degree of acetylation of hemicellulose and lignin severely affect the enzymatic hydrolysis of lignocellulosic biomass, so a pretreatment step is required before hydrolysis [45]. There are usually four categories of pretreatment such as chemical, physical, biological, and physicochemical which are used to overcome the complications listed above [46]. Organosolv is a pretreatment method, whereby the biomass is cooked at high temperature (100‒250 °C) in the presence of organic solvents (mainly ethanol), aimed at removing the lignin and hemicellulose into the liquid phase while producing a cellulose-rich solid fraction [47]. Lignin can easily be recovered from the liquid fraction as a solid with high purity, resulting in the generation of three relatively pure fractions. An important and advantageous feature of this method is the ability to recover the organic solvent by distillation, because of the low boiling point, and to use it again for pretreatment [18]. However, organosolv is effective for delignification of biomass but offers poor biomass deconstruction, while steam explosion is effective for the fractionation of biomass, so, in the current work, we used a hybrid organosolv—steam explosion method for the efficient fractionation and pretreatment of birch and spruce biomass [48].

**Fig. 2:**
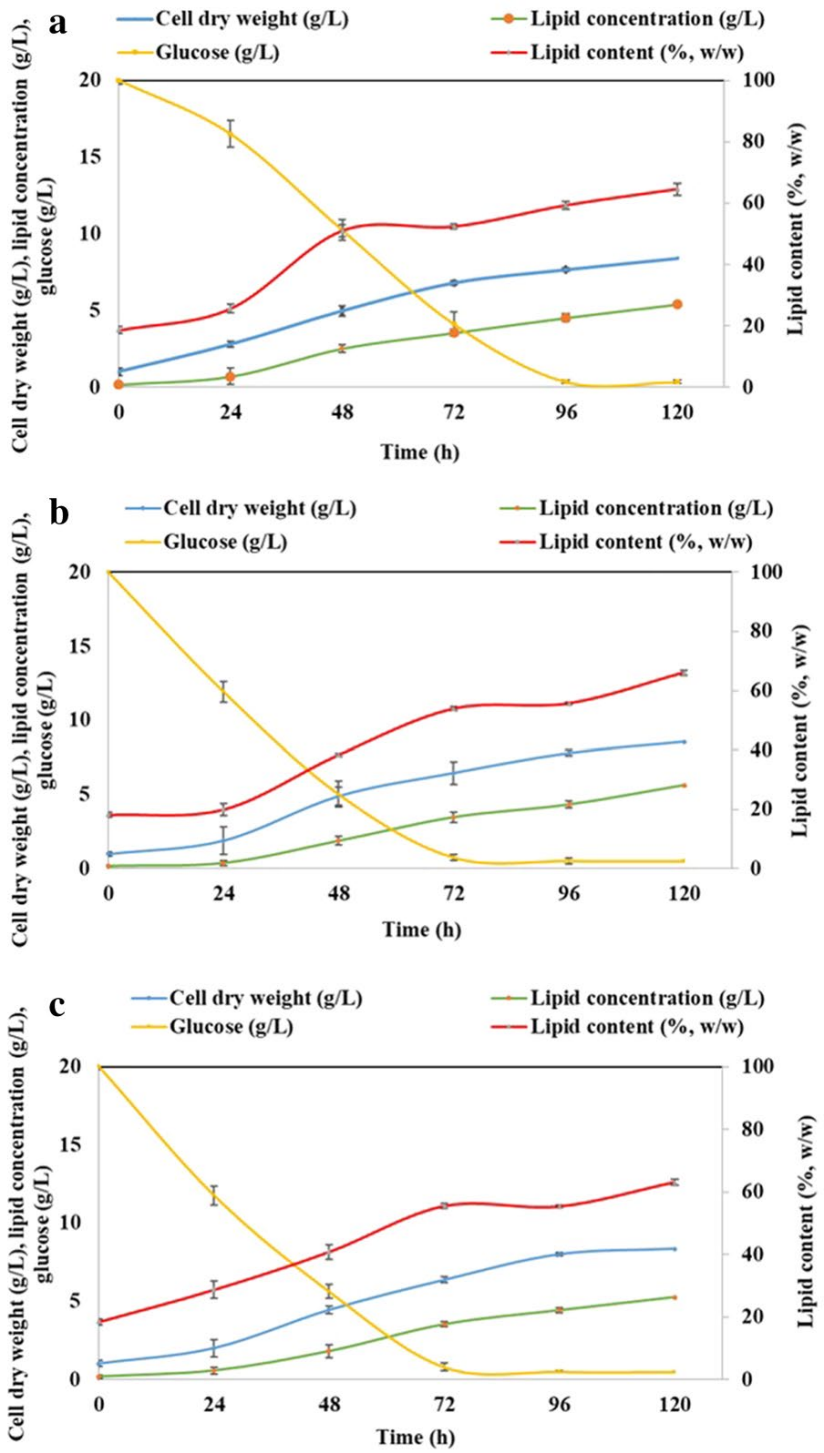
Batch cultivation of *A. protothecoides* in GSM, OPBH, and OPSH. Time course experiments for cell dry weight (g/L), total lipid concentration (g/L), lipid content (% w/w), and residual glucose in *A. protothecoides* grown for 120 h on **a** GSM; with a C/N ratio of 60, **b** OPBH; with a C/N ratio of 60 and **c** OPSH; with a C/N ratio of 60

During the current work, cellulose-rich pretreated solids from birch and spruce biomass were explored for the possibility of using the hydrolysates as a medium for growth and lipid accumulation in the oleaginous microalgae *A. protothecoides*, instead of GSM. For the production of the OPBH (organosolv-steam explosion pretreated birch hydrolysate) and OPSH (organosolv-steam explosion pretreated spruce hydrolysate), the pretreated solids were enzymatically hydrolyzed as described in Materials and methods. After the enzymatic hydrolysis, the concentration of glucose for OPBH and OPSH was 77.07 and 64.70 g/L, respectively. This is equivalent to 89 and 80.9% cellulose hydrolysis for OPBH and OPSH, respectively, demonstrating the effectiveness of the pretreatment in producing easily hydrolysable pretreated solids. Another important achievement of this treatment was that the hydrolysates were devoid of any inhibitors. The time course experiments for cell dry weight, total lipid concentration, lipid content, and residual glucose for *A. protothecoides* culture are presented in Fig. 2. Almost similar cell dry weight (8.56 ± 0.21 g/L) was obtained when cells were grown on OPBH for 120 h, whereas the cell dry weight was 8.40 ± 0.12 and 8.37 ± 0.13 g/L with GSM and OPSH, respectively (Table 3). The corresponding lipid content was 66.00 ± 0.33, 64.52 ± 0.53, and 63.08 ± 0.71% on a cell dry weight basis for OPBH, GSM, and OPSH, respectively (Table 3). The highest biomass productivity (1.71 ± 0.07 g/L day) and lipid productivity (1130 ± 24 mg/L day) were observed when this microalga was grown on OPBH (Table 3). The time course experiments for culture on OPBH, GSM, and OPSH showed no significant differences between the cultures. One possible explanation for this phenomenon is that it was mainly due to the pretreatment technique where hydrolysates contained pure glucose devoid of any inhibitors. At the end of the culture period (120 h), almost complete utilization of glucose was observed from the cultures grown on GSM, OPBH, and OPSH, respectively (Fig. 2). Oleaginous microalgae have the ability to use a carbon source present in the medium provided, irrespective of its origin and type [9, 10]. In contrast to our results, when the microalga *C. protothecoides* was grown on the enzymatic hydrolysates of sweet sorghum juice containing 10 g/L reducing sugar, the measured cell dry weight and lipid content were 5.10 g/L and 52.5% w/w, respectively, after 120 h of culture (Table 1). It was found that this microalga had approximately 36% higher lipid production when grown on enzymatic hydrolysates of sweet sorghum juice than when using glucose [25]. Similarly, *C. protothecoides* grown on cassava starch hydrolysate (containing 30 g/L glucose) and glucose (40 g/L) showed a cell dry weight of 15.8 and 10.7 g/L after 240 h of culture, and the corresponding values for lipid content were 26.5 and 30.7%, respectively [22]. In another study conducted by Xu et al. [21], the same species synthesized 3.74 and 3.92 g/L cell dry biomass when grown on glucose (10 g/L) and corn powder hydrolysate after 144 h of culture, with a corresponding lipid content of 54.7 and 55.3%, respectively. Mu et al. [20] suggested that *C. protothecoides* grown on sugarcane bagasse hydrolysate (SBH) performed better than glucose regarding cell growth and lipid accumulation under the same reducing sugar concentration. It produced 5.8 g/L of cell dry weight after 96 h when grown on SBH, whereas the cell dry weights were 4.23 and 2.34 g/L after 120 h with an artificial mixture of sugars and glucose, respectively. When the oleaginous microalga *Chlorella vulgaris* was grown on waste hydrolysate from *Cyperus esculentus*, it had 4.43 g/L biomass and 34.44% lipid content; the corresponding values were 1.85 g/L and 22.89%, respectively, when pure glucose (30 g/L) was used in the medium [23].

**Table 3:** Assessment of biomass and lipid accumulation in heterotrophically cultivated *A. protothecoides*

Parameters	GSM (C/N, 60)	OPBH (C/N, 60)	OPSH (C/N, 60)
Cell dry weight (g/L)	8.40 ± 0.12	8.56 ± 0.21	8.37 ± 0.13
Biomass productivity^a^ (g/L day)	1.68 ± 0.09	1.71 ± 0.07	1.67 ± 0.08
Total lipid concentration (g/L)	5.42 ± 0.27	5.65 ± 0.21	5.28 ± 0.17
Lipid content (%, w/w)	64.52 ± 0.64	66.00 ± 0.33	63.08 ± 0.71
Lipid productivity^a^ (mg/L day)	1084 ± 14	1130 ± 24	1056 ± 21

Quantitative estimation of cell dry weight (g/L), total lipid concentration (g/L), lipid content (% w/w), biomass productivity, and lipid productivity in *A. protothecoides* grown on GSM, OPBH, and OPSH

^a^The total biomass productivity and lipid productivity were calculated when the cell dry weight reached its highest value

The commercial aspect of biodiesel production from oleaginous microalgae in heterotrophic cultivation is limited by the cost of feedstocks, as this accounts for 60‒85% of the total production cost [16]. Researchers are interested in using lignocellulosic biomass for the cultivation of microalgae, but, till now, only agricultural residues and easily hydrolysable starch-based carbon sources have been used, such as corn powder, sweet sorghum juice, and cassava starch (Table 1), and the use of forest biomass for heterotrophic cultivation of microalgae has not yet appeared in the literature [49]. Woody lignocellulosic biomass obtained from forests remains an important feedstock for the heterotrophic cultivation of microalgae [16]. The forest residues such as top thin branches, trimmings, small trees, and un-merchantable wood are one of the largest available feedstocks on earth; they are often left over in the forest or used for the low-cost production of energy and heating by burning [18].

### Biochemical and morphological changes in *A. protothecoides* grown on various substrates

**Fig. 3:**
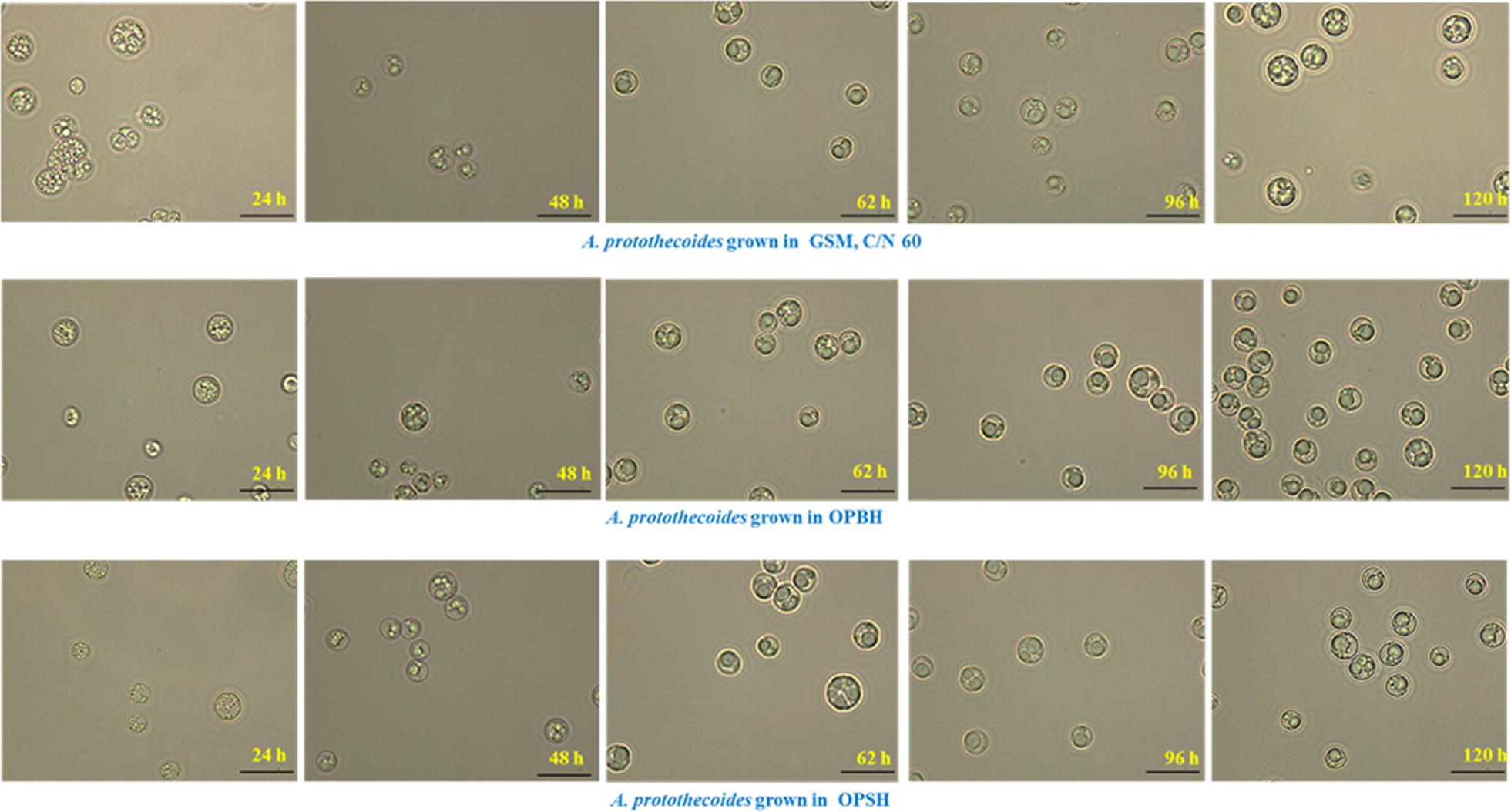
Microscopic analysis of microalgal growth. Representative images of different time intervals for the morphological analysis of *A. protothecoides* grown on GSM, OPBH, and OPSH (all with a C/N ratio of 60)

**Fig. 4:**
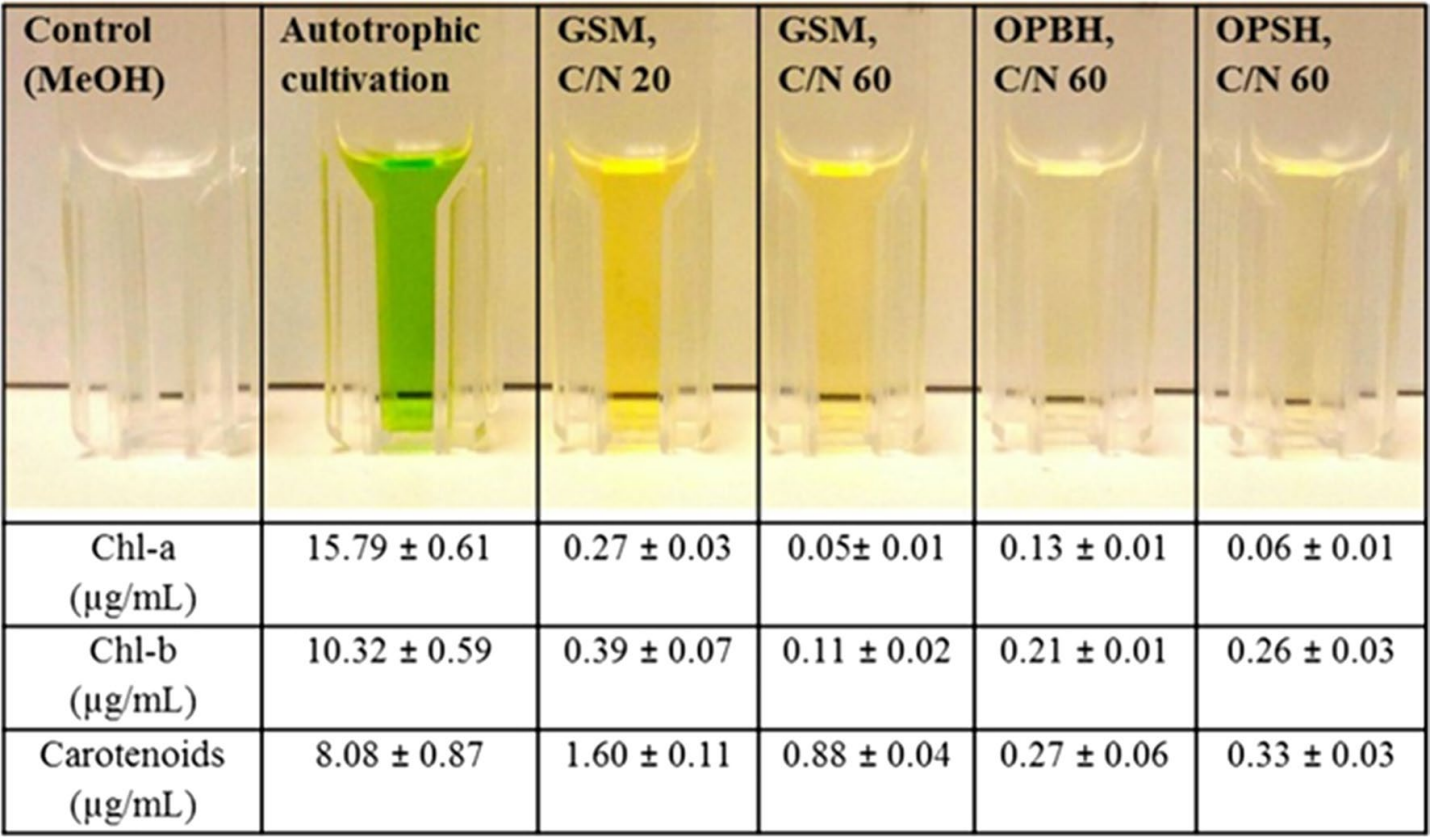
Analysis of pigments from autotrophically and heterotrophically cultivated microalga. Estimation of pigment composition obtained during the heterotrophic culture of *A. protothecoides* grown for 120 h on GSM, OPBH, and OPSH, (all with a C/N ratio of 60) and compared to its autotrophic mode of cultivation

Various changes are observed in the biochemical composition of algal cells, including changes in photosynthetic pigments, when microalgae are grown heterotrophically rather than by autotrophic culture [10]. The morphological variation of *A. protothecoides* grown in GSM, OPBH, and OPSH (with a C/N ratio of 60) was analyzed by light microscopy and images of different time intervals are presented in Fig. 3. This study showed that the morphological features of the cells cultivated in different medium (GSM, OPBH, and OPSH) were not significantly different. Some small growing colonies were observed during the initial growth phase (0–48 h), but all cells attained the similar size after glucose exhaustion from the medium (96–120 h). Carbon and nitrogen are interlinked through shared pathways to assimilate organic carbon by heterotrophic culture and generation of energy in mitochondria via the TCA cycle [10]. Nitrogen is the sole source for the chlorophyll and photosystem protein synthesis, so a drop in chlorophyll and carotenoids content is observed under nitrogen-limiting conditions [50]. It has already been reported that expression of the genes for chlorophyll metabolism, along with those for photosynthesis and carotenoid biosynthesis, is dramatically downregulated in heterotrophic microalgal cells relative to autotrophic cells of *C. protothecoides*, while expression of the genes for glycolysis, the TCA cycle, and fatty acid synthesis is up-regulated [25]. A high ratio of chlorophyll a to chlorophyll b and a high carotenoid/total chlorophyll ratio in microalgae under nitrogen-limiting conditions gave reduced light-harvesting complexes that help to induce lipid production and protect the cells against oxidative stress [50]. Chlorophyll a, chlorophyll b, and carotenoid concentrations were analyzed in *A. protothecoides* grown on GSM, OPBH, and OPSH, where cells grown autotrophically was used as a control to compare with heterotrophic growth (Fig. 4). The highest amounts of chlorophyll a (15.79 ± 0.61 µg/mL), chlorophyll b (10.32 ± 0.59 µg/mL), and carotenoids (8.08 ± 0.87 µg/mL) were observed in *A. protothecoides* that was grown autotrophically. The amount of pigments was almost double when this microalga was grown on GSM with a C/N ratio of 20, compared to when grown on GSM with a C/N ratio of 60. Similar findings were reported by Pancha et al. [51] where the microalga *Scenedesmus* sp. was grown under nitrogen limitation and sequential nitrogen starvation conditions, and showed reduced photosynthetic activity and total protein content of cells.

The importance of identifying the level of the pigments in oil feedstocks lies in the fact that pigments make the oil more susceptible to photo-oxidation, which further decreases the storage stability of the oil [52]. Moreover, the presence of pigments interferes with the transesterification reaction as well as the combustion of biodiesel, decreasing their efficiency [53]. Therefore, it is crucial to remove the chlorophyll from the microalgal oil feedstocks before processing them through transesterification reaction. There are several conventional methods already processed for the removal of chlorophyll from the oil feedstocks including physical absorption, oxidation treatment, and phosphoric acid degumming, with their use to further increase the overall production cost of biodiesel [54]. In this context, the present study of heterotrophically cultivated microalgae at high C/N ratio showed minimum amount of pigments compared to those grown autotrophically at low C/N ratio, avoiding the time-consuming and costly removal of the pigments prior to biodiesel formation.

### Estimation of the quantity of triacylglycerol (TAG) in total lipid extracted from A. *protothecoides* grown in GSM, OPBH, and OPSH, using TLC

Microalgae synthesize a diverse range of lipids (neutral lipids, glycolipids, and phospholipids) to perform various physiological functions. Glycolipids and phospholipids take part in the synthesis of the external membrane and the membranes of the chloroplast and the endoplasmic reticulum, while triacylglycerol is mainly neutral lipid stored in the form of lipid droplets in cellular compartments that are used to produce biodiesel by transesterification reaction. It has been reported that for various oleaginous microalgae grown under nitrogen-limiting conditions, the molecular mechanisms are shifted to accumulate large quantities of triacylglycerol instead of synthesizing cellular protein [55].

**Fig. 5:**
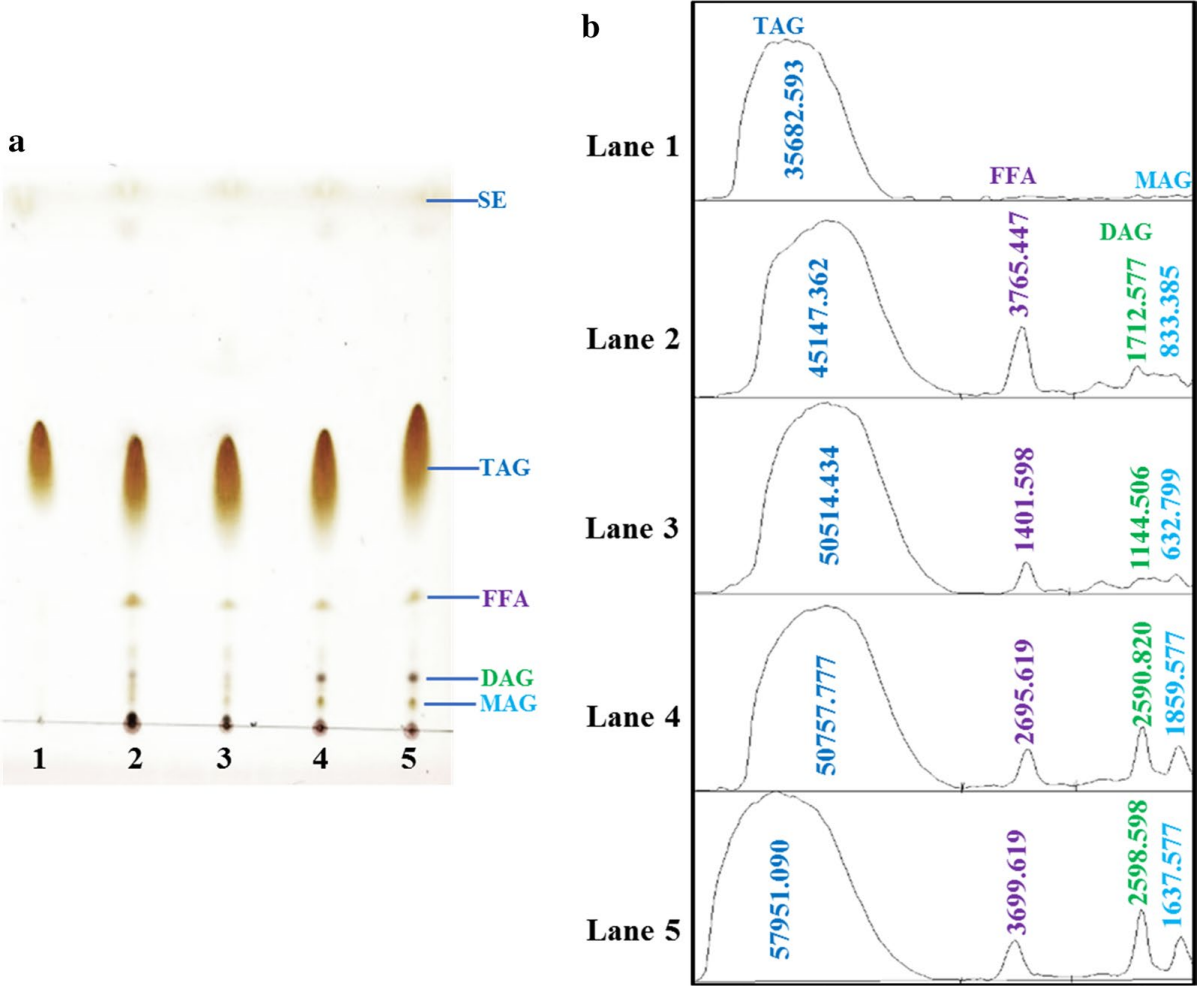
TLC analysis of extracted lipids. **a** Separation of total lipid extracted from *A. protothecoides* grown on GSM with a C/N ratio of 20 (lane 2), GSM with a C/N ratio of 60 (lane 3), OPBH with a C/N ratio of 60 (lane 4), and OPSH with a C/N ratio of 60 (lane 5), whereas glyceryl trioleate used as standard for TAG (lane 1). **b** Quantitative analysis of area corresponding to the optical density of each spot within a particular lane

There are various analytical methods such as HPLC, GC, GPC, 1H NMR, and NIR that are available for detection and identification of the lipids extracted. However, considering the relatively high cost of analysis and the time consumed using these methods, TLC is the best choice for detection of all the classes of lipids within a few hours. The availability of pre-coated plates makes it possible to obtain more reproducible results in the analysis of complex lipids within a short time. A TLC chromatogram of lipids extracted from *A. protothecoides* grown in GSM with a C/N ratio of 20 and a C/N ratio of 60, OPBH, and OPSH is presented in Fig. 5a, and optical densities of the corresponding areas of different spots are given in Fig. 5b. The amounts of all components of the lipids extracted were calculated from the corresponding areas represented in Fig. 5b. The data from lane 1 show the amount of triacylglycerol (TAG) when glyceryl trioleate (Sigma T7140) was used as standard (Fig. 5a). The highest amount of free fatty acid (FFA) was observed in lipid extracted from cells grown in GSM with a C/N ratio of 20, while the amounts of diacylglycerol (DAG) and monoacylglycerol (MAG) were highest in OPSH- and OPBH-grown cells (Fig. 5b). Oil feedstocks with a high amount of FFA are not suitable for conversion into biodiesel using an alkaline catalyst [56]. Oil feedstocks with high FFA content need to be esterified first with an acid catalyst to reduce the content of FFA in the feedstock, before transesterification with a basic catalyst to complete the reaction [57]. Feedstocks with a high amount of TAG are always a better choice for biodiesel production through transesterification, because, in a complete reaction, every mole of TAG produces three moles of biodiesel and one mole of glycerol [57]. According to the above observation, OPBH and OPSH are appropriate for the accumulation of high amounts of TAG, DAG, and MAG in a cellular compartment of a microalga.

### Estimation of fatty acid profile and biodiesel properties

The oleaginous microalga *C. protothecoides* usually synthesizes triacylglycerol in its cellular compartments, ranging from C_14_ to C_24_ when grown under heterotrophic conditions. TAG produced by *A. protothecoides* with GSM, OPBH, and OPSH were transmethylated into FAME, and the specific fatty acid profiles are summarized in Table 4. Altogether, ten different types of fatty acids including saturated (SFA), monounsaturated (MUFA), and polyunsaturated (PUFA) were detected. C_18:1_ and C_18:2_ were the major components of all three oils, which contributed approximately more than 80% to the total fatty acids. The fatty acid profile of *A. protothecoides* cells when grown on GSM with a C/N ratio of 60 showed the presence of mainly C_16:0_ (9.3%), C_18:0_ (2.85%), C_18:1_ (70%), C_18:2_ (13.6%), and C_18:3_ (1.74%), and included traces of C_14:0_, C_17:0_, C_17:1_, and C_20:1_ (Table 4). As discussed earlier, no significant differences were found in the lipid content of *A. protothecoides* when grown in GSM, OPBH, or OPSH, so no difference in their relative fatty acid contents was observed. Similar to the profile in the current work, *C. protothecoides* was found to synthesize a similar proportion of C_18:1_ when grown on various substrates such as molasses (67.95%) [26], rice straw hydrolysate (70.18%) [19], sugarcane juice hydrolysate [27], Jerusalem artichoke tuber hydrolysate (71.57%) [28], sweet sorghum juice (66.80%) [25], and cassava starch hydrolysate (71.25%) [24]. The FAME profile showed that the majority of the fatty acids were MUFA (monounsaturated fatty acids). It has been reported that the high quantity of PUFA (polyunsaturated fatty acids, mainly C_18:2_) is responsible for the integrity of the cell membrane in the lag phase of growth, while SFA (saturated fatty acids) (mainly C_18:0_) and MUFA (mainly C_18:1_) mainly accumulate in the early stationary phase of growth. C_18:0_ is converted to C_18:1_ by addition of double bonds by a desaturase in a later phase of growth [58], which results in a requirement for high quantities of oxygen, NADH, NADPH, and a substrate that finally prevents the accumulation of reactive oxygen species under stress conditions such as nitrogen limitation [59].

**Table 4:** Analysis of lipid profile of heterotrophically cultivated *A. protothecoides* by gas chromatography

Fatty acids	GSM (C/N, 60)		OPBH (C/N, 60)		OPSH (C/N, 60)	
Saturated fatty acid (SFA)						
(C_14:0_)	0.7	13.7%	0.7	13.51%	0.6	12.8%
(C_16:0_)	9.3		9.7		8.9	
(C_17:0_)	0.22		0.41		0.2	
(C_18:0_)	2.85		2.7		3.1	
Mono unsaturated fatty acid (MUFA)						
(C_16:1_)	0.26	70.54%	0.22	70.54%	0.22	69.74%
(C_17:1_)	0.1		0.17		0	
(C_18:1_)	70		70		69.5	
(C_20:1_)	0.18		0.15		0.2	
Poly unsaturated fatty acid (PUFA)						
(C_18:2_)	13.6	15.34%	13.8	15.27%	13.5	15.28%
(C_18:3_)	1.74		1.47		1.78	

Fatty acids of corresponding FAME profiles obtained after transesterification of lipids extracted from *A. protothecoides* grown on GSM, OPBH, and OPSH

In this work, an attempt was made to estimate significant biodiesel properties based on the fatty acid profile of the oleaginous microalgae grown in GSM, OPBH, or OPSH (Table 5). The chain length of the fatty acids, including the number and position of double bonds, severely affects the physiochemical properties of biodiesel, which must conform to criteria established by international standards such as ASTM 6751-3 (USA) and EN 14214 (Europe). The biodiesel standard EN 14214 is accepted for all the states of the European Committee [6]. LCSF is one of the most important characteristics of microbe-derived oil; this includes fatty acids where all the carbons of the fatty acid chains are totally saturated with H atoms, and this is directly correlated to the cetane number (CN), the CFPP, and the viscosity of biodiesel. A high LCSF number gives a high CN value, which in turn leads to reduced NOx emissions [60]. However, it has a negative effect on the CFPP, the viscosity, and the oxidative stability of biodiesel. The amount of LCSF was 2.36, 2.32, and 2.44 for the biodiesel obtained from GSM-, OPBH-, and OPSH-grown cells, respectively. Oxidative stability determines the shelf-life of biodiesel and it depends on the degree of unsaturation of the fatty acids. Biodiesel obtained from all the above-listed sources had more than 10 h of oxidative stability, thus satisfying the criteria stipulated by ASTM 6751-3 (minimum 3 h) and EN 14214 (minimum 6 h). In European countries, especially in the northern parts of Europe, the low-temperature operability of biodiesel is a significant problem due to gelling and crystallization in the fuel line and in the filters of the engine. In this respect, CFPP is an important criterion for measurement of the lowest temperature (in  °C) at which biodiesel can smoothly pass through a standardized filtration device in a specific time [5]. As listed in Table 5, all three biodiesel samples had low CFPP values of − 9.08, − 9.19, and − 8.81 °C, demonstrating that the biodiesel could operate under low-temperature conditions. No criteria for CFPP have been set up by USA or EU, due to variation in temperature across geographic regions and due to the time of year, and different counties and areas follow different standards. One of the most important properties of biodiesel is the CN value, which defines the ignition features of the fuel and is, therefore, correlated to engine performance such as noise generation and CO emissions [5]. The biodiesels obtained from GSM, OPBH, and OPSH (as listed in Table 5) had CN values of 52.93 52.94, and 53.25, which satisfied the lower limit according to ASTM 6751-3 (minimum 47) and EN 14214 (minimum 51). CN has both higher and lower limits stipulated, as a low value is associated with problems with starting of the engine at low temperature, emission of hydrocarbons, and high noise levels, while a high value gives a quick start without mixing with air, thus reducing fuel efficiency [61]. The data for kinematic viscosity show that all biodiesel types listed in Table 5 fall within a narrow range (3.5–5 mm^2^/s). Biodiesel has lower mass energy than diesel, due to having a high amount of oxygen. When the fatty acid chain length is increased (for a constant level of unsaturation), the heating value increases due to a decrease in the mass fraction of oxygen [57, 61]. All other properties listed in Table 5 also follow the regulations of ASTM 6751-3 and EN 14214.

**Table 5:** Assessment of biodiesel properties by empirical formulas

Biodiesel properties	Units	GSM; C/N 60	OPBH; C/N 60	OPSH; C/N 60	Biodiesel standards	
					ASTM D6751	EN 14214
					Limits	Limits
Long chain saturation factor	–	2.36	2.32	2.44	–	–
Oxidative stability, 110 °C	h	10.27	10.31	10.30	3 min	6 min
Density	g/cm^3^	0.86	0.86	0.86	–	0.86–0.90
Cold filter plugging point	°C	− 9.08	− 9.19	− 8.81	–	–
Cetane number	–	52.93	52.94	53.25	47 min	51 min
Kinematic viscosity	mm^2^/s	3.87	3.89	3.84	1.9–6.0	3.5–5
Saponification value	mg KOH/g-oil	198.43	199.11	196.88	0.50 min	0.50 min
Iodine value	mgI_2_/100 g	92.75	92.31	92.34	–	120 max
High heating value	MJ/kg	39.09	39.20	38.82	–	–

–, not reported; min, minimum; max, maximum

Estimation of biodiesel properties based on the fatty acid profiles of *A. protothecoides* grown on GSM, OPBH, and OPSH and comparison with the international standards

## Methods

### Strain and culture conditions

*Auxenochlorella protothecoides* SAG 211-7a was obtained from the culture collection of algae (SAG) at Göttingen University, Germany, and it was maintained at 16 °C on agar plates containing Bold’s basal medium (BBM). It was initially grown autotrophically and axenically in a photobioreactor (Multi-Cultivator MC 1000-OD; Photon Systems Instruments, Czech Republic) containing BBM and yeast extract (3.35 g/L) as nitrogen source under 18/6 h light/dark regimen (intensity of 43 μmol/m^2^ s) at 25 ± 1 °C. Aeration was provided by bubbling air through at normal pressure. For heterotrophic culture, cells were harvested from the photobioreactor by centrifugation, washed twice with sterile distilled water, and resuspended in 0.9% sterilized saline to obtain a cell density of 6.9–9.2 × 10^8^ cells/mL. For the preparation of inoculum, *A. protothecoides* was grown in 500-mL Erlenmeyer flasks containing 200 mL medium at 25 °C in an incubator in the dark with continuous shaking (180 rpm). BBM was used as basal medium supplemented with glucose (20 g/L) and yeast extract (3.35 g/L) was used as a source of nitrogen to achieve the desired C/N ratio. To determine the effect of the initial concentration of glucose on the biomass and lipid concentration of *A. protothecoides*, five different glucose concentrations (20, 40, 60, 80, and 100 g/L) were added with BBM in the GSM. The appropriate concentration of yeast extract was added to each flask to achieve a C/N ratio of 20. Optimization of the initial carbon concentration for maximum biomass was followed by optimization of lipid accumulation with various C/N ratios (20, 40, 60, 80, and 100) by varying the concentrations of yeast extract at the concentration of carbon (glucose) that was found to be optimal. After optimization of carbon and nitrogen concentrations for maximum biomass and lipid accumulation by *A. protothecoides*, OPBH- and OPSH-based media were used by adding appropriate volumes of OPBH and OPSH solutions to the basal medium (BBM) to achieve the necessary glucose concentration. Yeast extract was used as a nitrogen source and was added at a concentration that would give the desired C/N ratio. All glucose-based, OPBH-based, and OPSH-based media were adjusted to pH 6.8 before autoclaving. Each flask was inoculated with 10% of seed culture and culture was performed in an orbital shaker (180 rpm) at 25 °C in the dark. Samples were taken at regular intervals to determine the cell density, lipid content, and reducing sugar concentration.

### Preparation and enzymatic hydrolysis of organosolv-steam explosion pretreated birch and spruce

Silver birch (*Betula pendula* L.) and Norway spruce (*Picea abies* L.) chips milled at less than 1 mm in size in a Retch SM 300 knife mill (Retsch GmbH, Haan, Germany) were pretreated with a hybrid organosolv-steam explosion pretreatment method that was previously developed by our group [48]. In brief, the pretreatment conditions were as follows: birch was treated at 200 °C with 60% v/v ethanol and 1% w/w_biomass_ of H_2_SO_4_ for 15 min, and spruce was treated at 200 °C with 52% v/v ethanol and 1% w/w_biomass_ of H_2_SO_4_ for 30 min. At the end of the pretreatment, the solids were separated from the liquid by vacuum filtration, washed with ethanol, and air-dried until further use. The composition (w/w) of the pretreated birch solids was 77.9% cellulose, 8.9% hemicellulose, and 7% lignin [48]; that of the spruce solids was 72% cellulose, 4% hemicellulose, and 15.4% lignin.

Enzymatic hydrolysis of pretreated birch and spruce biomass took place in 500-mL Erlenmeyer flasks containing 100 g of 10% w/w biomass solution in 50-mM citrate–phosphate buffer of pH 5. Hydrolysis was performed at 50 °C for 48 h with mixing at 180 rpm. The commercial enzyme solution Cellic CTec2 (Novozymes A/S, Bagsværd, Denmark) was used at a concentration equal to 20 FPU/g of solids. At the end of enzymatic hydrolysis, the solution was centrifuged to separate the remaining solids from the liquid, and the obtained hydrolysate was used as the carbon source for algal cultivation.

### Estimation of cell growth, cell dry weight (g/L), and biomass productivity, P (g/L day)

Cell growth was monitored by optical density measurement at 680 nm using UV/visible spectrophotometry. For cell dry weight measurement, 5 mL of culture broth was transferred to a pre-weighed tube and centrifuged at 8000 rpm for 5 min. The pellet obtained was washed and centrifuged twice with distilled water to remove medium components, whereas the supernatant was used for sugar determination. After rinsing, the pellet was dried at 50 °C to constant weight and cooled down at room temperature in a desiccator prior to weighing. Cell dry weight (g/L) was estimated gravimetrically. The volumetric biomass productivity, P (g/L day), was calculated using the following equation:

$$P = \frac{{\left( {X2 - X1} \right)}}{{\left( {t2 - t1} \right)}},$$

where *X*1 and *X*2 were the cell dry weights (g/L) on days *t*1 (start point of cultivation) and *t*2 (endpoint of cultivation), respectively.

### Determination of total lipid concentration (g/L), lipid content (%, w/w), and lipid productivity (mg/L day)

The dried samples from the 5 mL of culture broth were used for lipid extraction purposes. The dried microalgal cells were crushed with a mortar and pestle to fine powder and extracted using a chloroform:methanol (2:1) mixture overnight at room temperature, with constant shaking. The slurry was filtered using a 0.22-μm filter and the solvent containing lipid was transferred to pre-weighed glass vials. The glass vials were dried under vacuum and weighed to estimate the total lipid concentration (g/L). The lipid content (%, w/w) based on cell dry weight was measured using the following equation:

$$Y = \frac{{\rm TL}}{{\rm CDW}},$$

where *Y* was the lipid content (%, w/w), and TL and CDW were the total lipid concentration (g/L) and the cell dry weight concentration (g/L), respectively.

The lipid productivity (mg/L day) was calculated using the following equation:

$$P = \frac{{Y \times {\text{Biomass}}\; {\text{productivity}}}}{100},$$

where *P* was the lipid productivity and *Y* was the lipid content (%. w/w).

### Determination of residual sugar

The amount of residual sugar during the time course of the experiment was analyzed by HPLC with a refractive index detector and a Biorad Aminex HPX-87P column (Bio-Rad, Hercules, CA, US). The column was maintained at 85 °C and ultrapure water was used as the mobile phase at a flow rate of 0.6 mL/min. The sugar consumption (%) was calculated by the following equation:

$$C = \frac{{{\text{St}}1 - {\text{St}}2}}{{{\text{St}}1}} \times 100,$$

where *C* was the amount of sugar consumption, St1 was the amount of initial sugar added (g/L), and St2 was the residual sugar left at each sampling time.

### Biochemical and morphological analysis including accumulation of lipid droplets in microalgal cells

To analyze the morphological changes of *A. protothecoides* grown in GSM, OPBH, and OPSH, 10 µL of culture was drawn at different time intervals and pelleted. After washing three times with 0.9% w/w saline, the cells were visualized by compound light microscopy (Olympus, Germany).

The photosynthetic pigments (chlorophyll a, chlorophyll b, and carotenoids) from autotrophically and heterotrophically grown cultures were determined on the fourth day (i.e., the early stationary phase) of cultivation. Cells (in 2 mL of culture broth) were harvested and washed with distilled water; then, methanol (2 mL, 99.9%) was added to the pellets before incubation at 45 °C for 24 h, followed by centrifugation to remove cell debris. The supernatant (extraction volume of 800 μL) was used to measure the absorbance at 665.2, 652.4, and 470 nm with a UV/visible spectrophotometer and the amounts of pigment were determined using the following equations:

$${\text{Chlorophyll }}\;{\text{a}} \;\left( {{\text{Chl}}\; {\text{a}}; \;{{\upmu{\text{g}}} \mathord{\left/ {\vphantom {{\upmu{\text{g}}} {\text{mL}}}} \right. \kern-0pt} {\text{mL}}}} \right) = 16.72\;A_{665.2} - 9.16 \;A_{652.4}$$

$${\text{Chlorophyll}}\; {\text{b}} \;\left( {{\text{Chl}}\;{\text{b}}; \;{{\upmu{\text{g}}} \mathord{\left/ {\vphantom {{\upmu{\text{g}}} {\text{mL}}}} \right. \kern-0pt} {\text{mL}}}} \right) = 34.09 \;A_{652.4} {-} 15.28 \;A_{665.2}$$

$${\text{Carotenoids}}\; \left( {{{\upmu{\text{g}}} \mathord{\left/ {\vphantom {{\upmu{\text{g}}} {\text{mL}}}} \right. \kern-0pt} {\text{mL}}}} \right) = 1000 \;A_{470} - \left( {1.63 {\text{Chl}} \;{\text{a}} - 104.9 {\text{Chl}}\; {\text{b}}} \right)/221 ,$$

where *A* was the absorbance at a particular wavelength [51].

### Analysis of neutral lipids in extracted lipids by TLC, fatty acid profile by GC, and estimation of biodiesel properties

To estimate the amount of triacylglycerol in the total lipids extracted, samples (2 µL) were spotted on silica gel G-60 (0.25-mm thick) on an F254 TLC plate (Merk, Germany) as explained in a previous protocol [62]. Briefly, the chromatograph was run to 8 cm from the origin with hexane:diethyl ether:acetic acid (85:15:1, v/v/v) using glyceryl trioleate (Sigma cat. T7140) as standard. After drying in air, it was stained with methanolic MnCl_2_ solution (0.63-g MnCl_2_·4H_2_O, 60-mL water, 60-mL methanol, and 4-mL concentrated sulfuric acid), dried, and heated to 120 °C for 15 min. The stained plate was scanned with a CanoScan LiDE 210 scanner and the image obtained was processed with the Image J 1.48a software for measurement of optical density in the areas of different spots. The total lipid extracted (10 mg) was transesterified by mixing with acid catalyst (8 mL of 6% methanolic H_2_SO_4_) in a Teflon-coated screw-cap tube. The mixture was kept in a water bath at 60 °C for 2 h with gentle shaking. The slurry was cooled at room temperature, followed by addition of n-hexane (2 mL) and water (1 mL). The FAME (fatty acid methyl ester) was collected in the n-hexane layer after centrifugation. The FAMEs were analyzed by gas chromatography (Varian CP-3800; Agilent Technologies, Santa Clara, CA, USA) using a capillary column (WCOT-fused silica 100-m × 0.25-mm coating, CPSIL 88; Agilent Technologies) under operating conditions that have previously been reported [63]. Estimation of biodiesel properties was done using the following derived empirical formulae [6]:

1 $${\text{Long - chain saturation factor}}\;\left( {{\text{LCSF}}; \, \% {\text{wt}}} \right)\; = \left( {0.1*{\text{C}}_{16} } \right) + \left( {0.5*{\text{C}}_{18} } \right)$$

2 $${\text{Cold filter plugging point }}\left( {{\text{CFPP}}; \, ^\circ {\text{C}}} \right) = \left( {3.417*{\text{LCSF}}} \right){-}16.477$$

3 $${\text{Cetane number}}\;\left( {\text{CN}} \right) = {{\left( {46.3 + 5458} \right)} \mathord{\left/ {\vphantom {{\left( {46.3 + 5458} \right)} {\text{SV}}}} \right. \kern-0pt} {\text{SV}}}{-}\left( {0.255*{\text{IV}}} \right)$$

4 $${\text{Oxidative stability }}\left( {{\text{OS}};{\text{ h}}} \right) = {{117.9295} \mathord{\left/ {\vphantom {{117.9295} {\left( {{{\text{w}} \mathord{\left/ {\vphantom {{\text{w}} {\text{w}}}} \right. \kern-0pt} {\text{w}}}\% {\text{ C}}_{18:2} + {{\text{w}} \mathord{\left/ {\vphantom {{\text{w}} {\text{w}}}} \right. \kern-0pt} {\text{w}}}\% {\text{ C}}_{18:3} } \right) + 2.5905}}} \right. \kern-0pt} {\left( {{{\text{w}} \mathord{\left/ {\vphantom {{\text{w}} {\text{w}}}} \right. \kern-0pt} {\text{w}}}\% {\text{ C}}_{18:2} + {{\text{w}} \mathord{\left/ {\vphantom {{\text{w}} {\text{w}}}} \right. \kern-0pt} {\text{w}}}\% {\text{ C}}_{18:3} } \right) + 2.5905}}$$

5 $${\text{Saponification value}}\;\left( {{\text{SV}};{\text{ mg KOH}}} \right) = \sum {560 \, {{\left( {\% {\text{ FA}}} \right)} \mathord{\left/ {\vphantom {{\left( {\% {\text{ FA}}} \right)} {\text{Mi}}}} \right. \kern-0pt} {\text{Mi}}}}$$

6 $${\text{Iodine value}}\;\left( {{\text{IV}}; \, {{{\text{gI}}_{2} } \mathord{\left/ {\vphantom {{{\text{gI}}_{2} } {100\,{\text{g}}}}} \right. \kern-0pt} {100{\text{g}}}}} \right) = \sum {254{\text{DB}}} *{{\% {\text{FA}}} \mathord{\left/ {\vphantom {{\% {\text{FA}}} {\text{Mi}}}} \right. \kern-0pt} {\text{Mi}}}$$

7 $${\text{DU}}\left( \% \right) = {\text{MUFA}} + \left( {2*{\text{PUFA}}} \right)$$

8 $${\text{High heating value}}\;\left( {{\text{HHV}}; \, {{\text{MJ}} \mathord{\left/ {\vphantom {{\text{MJ}} {\text{Kg}}}} \right. \kern-0pt} {\text{Kg}}}} \right) = 49.43{-}0.041\left( {\text{SV}} \right){-}0.015\left( {\text{IV}} \right)$$

9 $${\text{Kinematic viscosity }}\left( {{\text{KV}}_{{(\upnu{\text{i}})}} ;\; 40\;^\circ {\text{C in }}{{{\text{mm}}^{ 2} } \mathord{\left/ {\vphantom {{{\text{mm}}^{ 2} } {\text{s}}}} \right. \kern-0pt} {\text{s}}}} \right) \, = \, \left( { - 1 2. 50 3+ 2. 4 9 6} \right)\, * \,{ \ln }\left( {\sum {\text{Mi}} } \right){-}0. 1 7 8*\sum {\text{DB}}$$

10 $${\text{Density}}\left( {\rho ; \, 20\;^\circ {\text{C in }}{{\text{g}} \mathord{\left/ {\vphantom {{\text{g}} {\text{cm}}}} \right. \kern-0pt} {\text{cm}}}} \right) = {{\left( {0.8463 + 4.9} \right)} \mathord{\left/ {\vphantom {{\left( {0.8463 + 4.9} \right)} {\sum {{\text{Mi}} + 0.0118} }}} \right. \kern-0pt} {\sum {{\text{Mi}} + 0.0118} }}*\sum {\text{DB}} ,$$

where Mi was the molecular weight of each fatty acid, DB was the number of double bonds, FA was the fatty acid content (%), MUFA was the monounsaturated fatty acid content, and PUFA was the polyunsaturated fatty acid content.

### Statistical analysis

In this study, all experiments were conducted in triplicates. The data were expressed as mean ± standard deviation and were analyzed with one-way analysis of variance (ANOVA) using Microsoft Office Excel 2016, with *p* values of < 0.05 being regarded as significant.

## Conclusion

There is an increasing research interest in the use of low-cost renewable resources (such as lignocellulosic biomass) for the cultivation of microalgae, with the purpose of producing lipids for biodiesel production. Although various sources of plant biomass have already been tried in the literature, wood biomass is an underexploited resource that, to the best of our knowledge, has not been used for the growth of microalgae. In this work, we wanted to develop a novel approach for biodiesel production using a hybrid organosolv-steam explosion pretreated birch and spruce hydrolysates and heterotrophic growth of *A. protothecoides*. The hybrid pretreatment method used in this study allowed the efficient fractionation of spruce and birch biomass along with production of pretreated solids with high cellulose and low lignin content that could also be applicable for other non-edible lignocellulosic biomasses. This microalga, when grown in OPBH or OPSH, synthesized high quantities of lipids (66.00 ± 0.33 and 63.08 ± 0.71%, w/w, respectively) and, to the best of our knowledge, this is the first time that the use of wood hydrolysates for the culture of microalgae has been described. Moreover, the FAME profiles of biodiesel obtained after growth on OPBH or OPSH satisfy the criteria set up by ASTM 6751-3 and EN 14214 for use as transportation fuel.
